# Neuropathological hallmarks of fetal hydrocephalus linked to *CCDC88C* pathogenic variants

**DOI:** 10.1186/s40478-021-01207-5

**Published:** 2021-06-06

**Authors:** Florent Marguet, Myriam Vezain, Pascale Marcorelles, Séverine Audebert-Bellanger, Kévin Cassinari, Nathalie Drouot, Pascal Chambon, Bruno J. Gonzalez, Arie Horowitz, Annie Laquerriere, Pascale Saugier-Veber

**Affiliations:** 1 UNIROUEN, INSERM U1245, Rouen University Hospital, Department of Pathology, Normandie Univ, 76000 Rouen, France; 2 UNIROUEN, Inserm U1245 and Rouen University Hospital, Department of Genetics and Reference Center for Developmental Disorders, Normandy Center for Genomic and Personalized Medicine, Normandie Univ, 76000 Rouen, France; 3 UBO EA 4685 LIEN and Brest University Hospital, Pathology Department, Brest Univ, 29609 Brest, France; 4 Department of Genetics, Brest University Hospital, 29200 Brest, France; 5 UNIROUEN, INSERM U1245, Normandy Centre for Genomic and Personalized Medicine, Normandie Univ, 76000 Rouen, France; 6 Pathology Laboratory, Pavillon Jacques Delarue, CHR, 1 rue de Germont, 76031 Rouen Cedex, France

**Keywords:** *CCDC88C* pathogenic variants, Autosomal recessive inheritance, Foetal hydrocephalus, Neuropathology, Multiple ependymal malformations, Choroid plexus hydrops, Neural tube defect, Renal agenesis, Diaphragmatic defect, Planar cell polarity

## Abstract

The prevalence of congenital hydrocephalus has been estimated at 1.1 per 1000 infants when including cases diagnosed before 1 year of age after exclusion of neural tube defects. Classification criteria are based either on CSF dynamics, pathophysiological mechanisms or associated lesions. Whereas inherited syndromic hydrocephalus has been associated with more than 100 disease-causing genes, only four genes are currently known to be linked to congenital hydrocephalus either isolated or as a major clinical feature: *L1CAM, AP1S2, MPDZ* and *CCDC88C*. In the past 10 years, pathogenic variants in *CCDC88C* have been documented but the neuropathology remains virtually unknown. We report the neuropathology of two foetuses from one family harbouring two novel compound heterozygous pathogenic variants in the *CCDC88C* gene: a maternally inherited indel in exon 22, c.3807_3809delinsACCT;p.(Gly1270Profs*53) and a paternally inherited deletion of exon 23, c.3967-?_c.4112-?;p.(Leu1323Argfs*10). Medical termination of pregnancy was performed at 18 and 23 weeks of gestation for severe bilateral ventriculomegaly. In both fetuses, brain lesions consisted of multifocal atresia-forking along the aqueduct of Sylvius and the central canal of the medulla, periventricular neuronal heterotopias and choroid plexus hydrops. The second fetus also presented lumbar myelomeningocele, left diaphragmatic hernia and bilateral renal agenesis. *CCDC88C* encodes the protein DAPLE which contributes to ependymal cell planar polarity by inhibiting the non-canonical Wnt signaling pathway and interacts with MPDZ and PARD3. Interestingly, heterozygous variants in *PARD3* result in neural tube defects by defective tight junction formation and polarization process of the neuroepithelium. Besides, during organ formation Wnt signalling is a prerequisite for planar cell polarity pathway activation, and mutations in planar cell polarity genes lead to heart, lung and kidney malformations. Hence, candidate variants in *CCDC88C* should be carefully considered whether brain lesions are isolated or associated with malformations suspected to result from disorders of planar cell polarity.

**Supplementary Information:**

The online version contains supplementary material available at 10.1186/s40478-021-01207-5.

## Introduction

Congenital hydrocephalus affects 4.65 per 10,000 births and its prevalence has been estimated at 1.1 per 1000 infants when including cases diagnosed before 1 year of age in the absence of other extrinsic causes and after exclusion of neural tube defects [16, 20]. Several classifications are recognized based on CSF dynamics (communicating *vs.* non-communicating; non-obstructive *vs.* obstructive), pathophysiological mechanisms (developmental *vs.* acquired) or associated lesions (syndromic *vs.* non-syndromic; associated with other major abnormalities *vs.* with brain lesions only) [15]. Whereas syndromic hydrocephalus has been associated with more than 100 disease-causing genes, only four genes are currently known to be linked to congenital hydrocephalus as isolated or as the major clinical feature: *L1CAM, AP1S2, MPDZ* and *CCDC88C*. Pathogenic *L1CAM* variants are responsible for a wide phenotypic spectrum, X-linked hydrocephalus with stenosis of the aqueduct of Sylvius (AS) being the most common genetic form, with a prevalence of 1:30,000 and accounting for approximately 5–10% of males with non-syndromic congenital hydrocephalus. L1CAM is a neuronal adhesion molecule of the immunoglobulin superfamily which plays major roles in intercellular adhesion, neuronal cell survival and migration, growth cone morphology and neurite outgrowth, axonal guidance and fasciculation, synaptogenesis and myelination [1]. Pathogenic *AP1S2* variants have been linked to Pettigrew syndrome characterized by intellectual disability with prominent basal ganglia iron deposition or calcification and variable severity of hydrocephalus. AP1S2 is a subunit of the AP1 adaptin protein complex, one of the major regulators of lysosomal protein sorting involved in clathrin-coated vesicle assembly and transport of proteins between the trans-Golgi network and lysosomes [14]. More recently, deleterious variants in *MPDZ* and *CCDC88C* genes were shown to be responsible for non-syndromic autosomal recessive congenital hydrocephalus. MPDZ, named alternatively MUPP1, is a modular scaffold protein consisting of 13 PDZ and one L27 domains localized to apical junction complexes [17]. The pathophysiology of *MPDZ*-linked hydrocephalus has been attributed to hyperpermeability of the choroid plexus epithelial cells in mice [21]. *CCDC88C* encodes the segment polarity protein disheveled homolog (Dvl)-binding protein DAPLE, a guanine exchange factor trimeric G proteins [2]. DAPLE contains a PDZ-binding motif at its C-terminus which contributes to ependymal cell planar polarity by inhibiting the non-canonical Wnt signaling pathway through its interaction with Dishevelled, leading to Rho-ROCK and Rac-JNK activation [10, 19]. In the past 10 years, pathogenic variants in *CCDC88C* have been documented but the neuropathology of the disease remains virtually unknown [4, 6, 13, 20]. We report herein the prenatal phenotype and neuropathological lesions in two fetuses from one family, which harboured two novel compound heterozygous variants in the *CCDC88C* gene.

## Case presentation

**Fig. 1:**
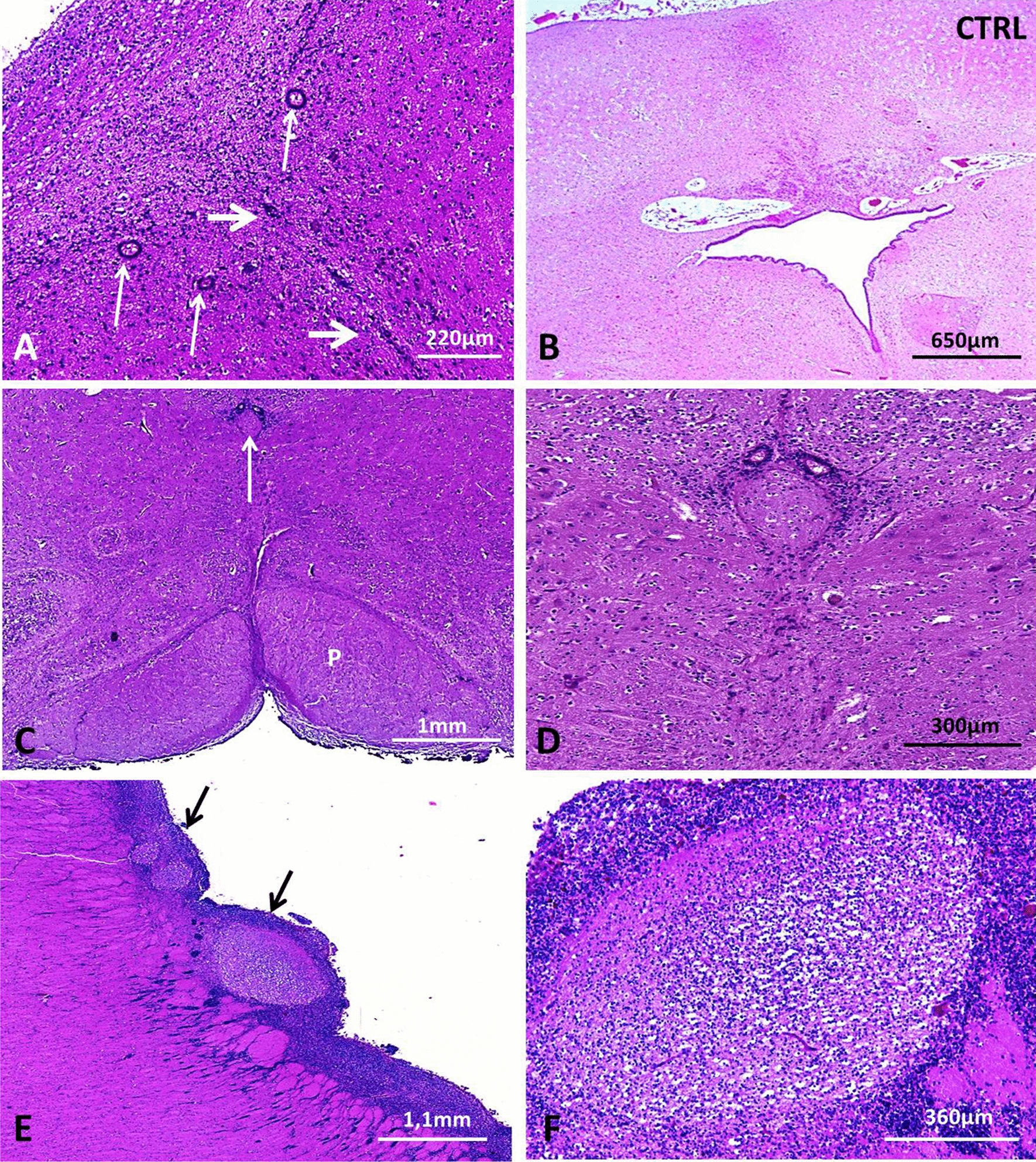
Main neuropathological hallmarks of hydrocephalus linked to *CCDC88C* pathogenic variants. **a** Coronal section through the mesencephalon reveals AS atresia in the first case (thick arrows) associated with small- and medium-sized channels lined by ependymal cells (thin arrows) [H&E, OM × 100)]. **b** By comparison with a normal aqueduct of Sylvius [H&E, OM × 100)]. **c** Identical lesions (arrow) observed in the second case at the level of the central canal of the medulla the AS [H&E, OM × 25]. **d** With, at higher magnification, presence of several small ependymal channels [H&E, OM × 100]. **e** Periventricular nodular heterotopias of various size (thick arrows) [H&E, OM × 25]. **f** With at higher magnification, a lower cell density [H&E, OM × 100)]. H&E: Hematoxylin and eosin stain; OM: original magnification; P: pyramids

**Fig. 2:**
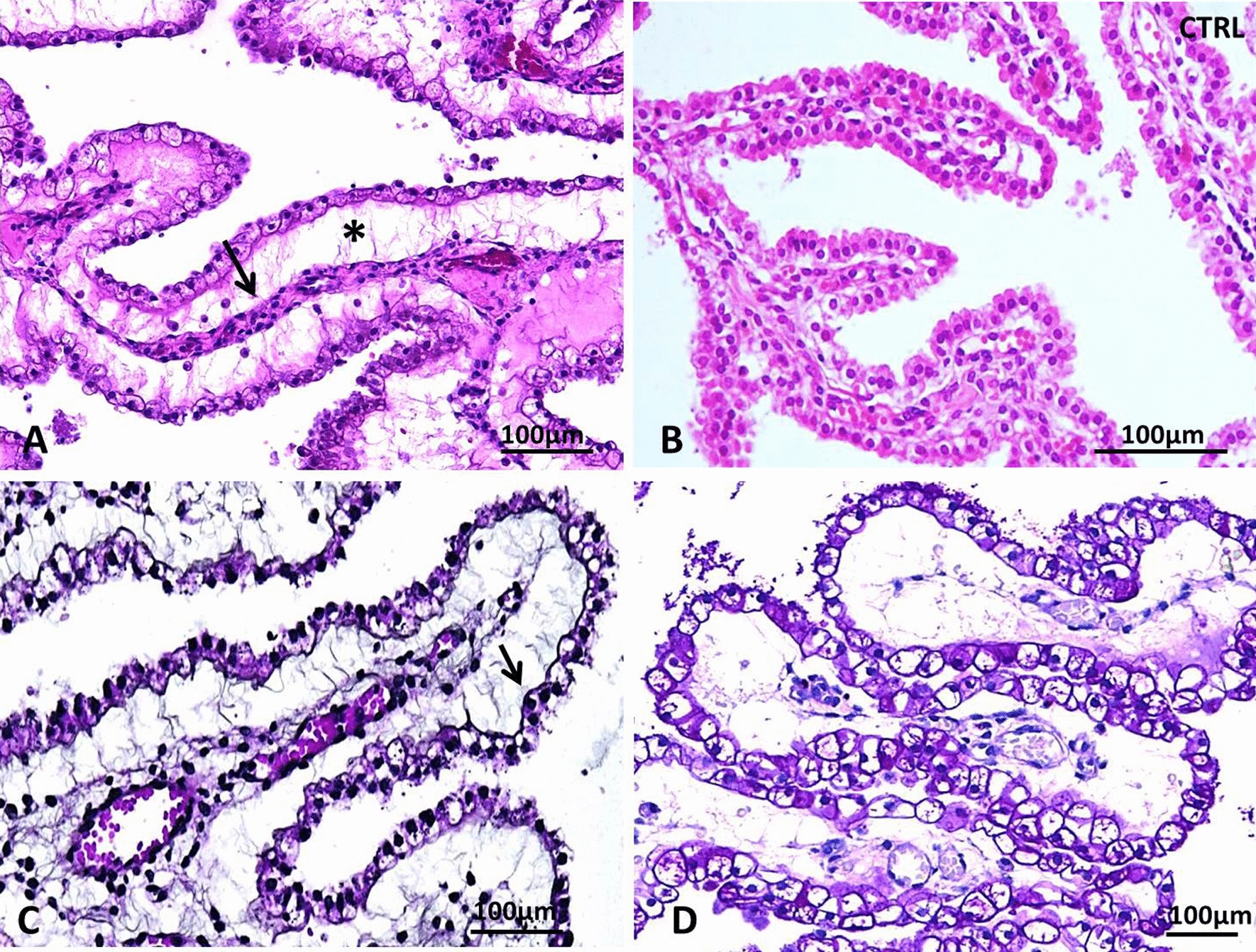
Main neuropathological hallmarks of hydrocephalus linked to *CCDC88C* pathogenic variants. **a** Accumulation of fluid (asterisk) under the choroid epithelium causing collapse of the villus mesenchymatous core which contains collapsed vessels (arrow) [H&E, OM × 200]. **b** Compared with the normal choroid villus morphology in an age- matched control [H&E, OM × 200]. **c** Without disruption of the basal lamina as evidenced by Jones’ silver impregnation method [H&E, OM × 200]. **d** Covered by epithelial cells still containing glycogen [PAS, OM × 200]. H&E: Hematoxylin and eosin stain; OM: original magnification; PAS: periodic Schiff staining

**Fig. 3:**
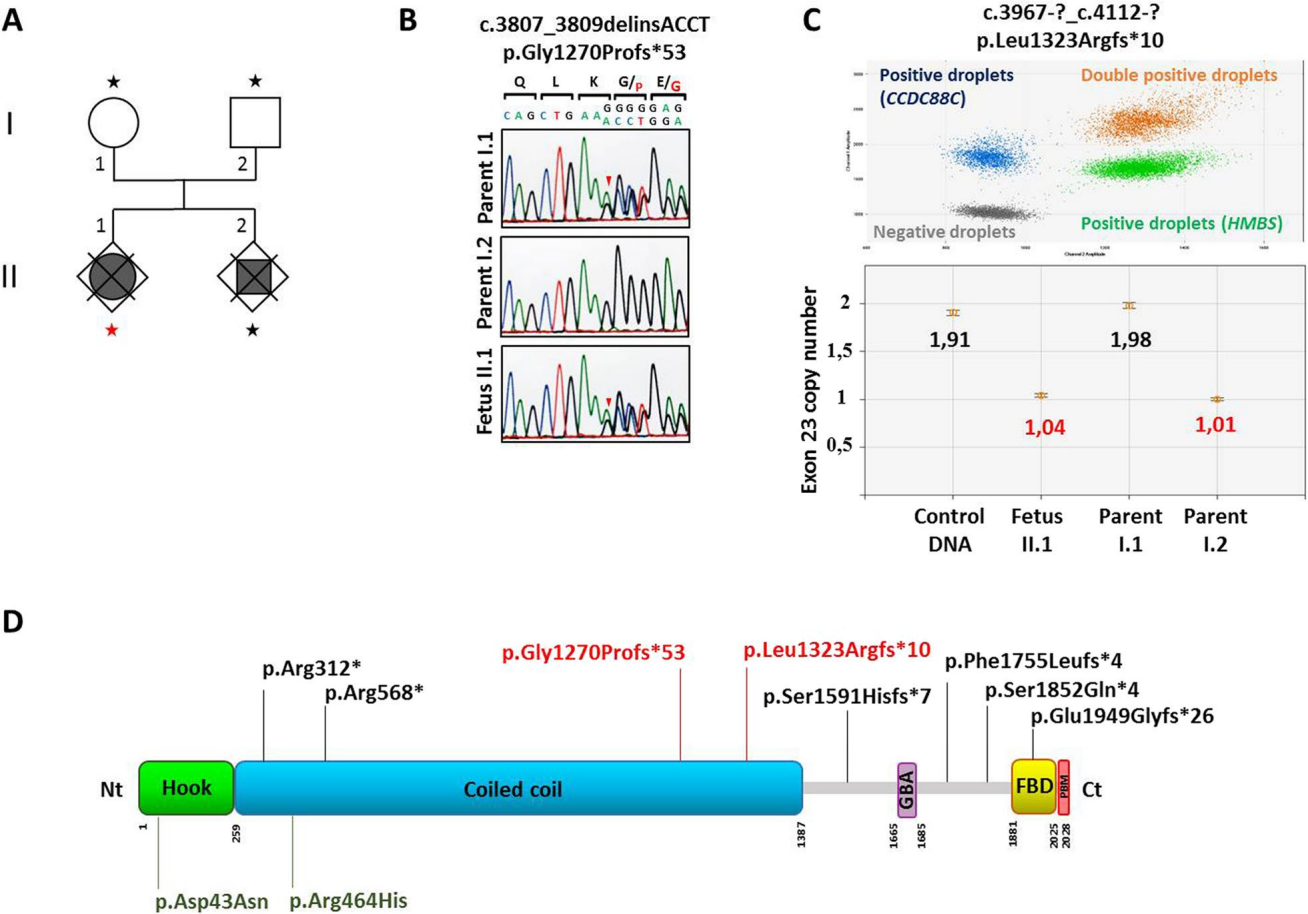
Identification of the two compound heterozygous variations in the *CCDC88C* gene. **a** Pedigree structure of the family. Targeted NGS sequencing of a panel including *L1CAM*, *MPDZ* and *CCDC88C* was performed in fetus II.1 (red star). Whole exome sequencing (WES) was performed in fetus II.2 and his parents (I.1 and I.2; black stars). **b** Targeted NGS sequencing and WES identified a heterozygous frameshift variant in the *CCDC88C* gene, c.3807_3809delinsACCT; p.(Gly1270Profs*53)**,** which was shown to be maternally inherited by Sanger sequencing of the fetuses and their parents. **c** Targeted NGS sequencing and WES also identified a heterozygous deletion of *CCDC88C* exon 23 (c.3967-?_c.4112-?; p.Leu1323Argfs*10), which was shown to be paternally inherited and was confirmed by a relative quantification ddPCR assay of fetus II.1 and her parents**.** Top: Representative result of the ddPCR assay, using of the *CCDC88C* gene (primers located in the exon 23, *blue droplets*) compared to a reference housekeeping gene (*HMBS* gene, *green droplets*). Bottom: Quantification of copy number in a control DNA (target, 1358 copies/μL; reference, 1426 copies/μL), fetus II.1 (target, 551 copies/μL; reference, 1057 copies/μL), parent I.1 (target, 1011 copies/μL; reference, 1019 copies/μL), and parent II.2 (target, 447 copies/μL; reference, 894 copies/μL). **d** Schematic representation of DAPLE protein organization. DAPLE contains a Hook domain, a Gα binding and activating domain (GBA), a coiled coil region, a frizzled binding domain (FBD) and a carboxy-terminal PDZ binding motif (PBM). The compound heterozygous variants identified in this study (in red) were localized in the coiled-coil domain. Published homozygous loss-of-function variants are depicted in black whereas heterozygous gain-of-function variations are represented in green (2,8–11). Nt: amino-terminal; Ct: carboxy-terminal

A 31-year-old woman, gravida I, para 0, underwent routine ultrasonography (US) at 22 weeks of gestation (WG), which revealed macrocephaly (head circumference >> 97th percentile) with severe bilateral ventriculomegaly, but with no other associated brain, visceral or growth parameter abnormalities. Based on these findings, a medical termination of the pregnancy (TOP) was achieved at 23 WG in accordance with the French law. Nine months later, TOP was performed at 18 WG after the discovery of similar brain lesions on US, associated with growth restriction and myelomeningocele. Chromosomal analysis revealed a normal karyotype, 46, XX and 46, XY respectively. CGH analysis was normal. The unrelated parents were in good health and there was no particular medical family history. At autopsy (Additional file 1), both fetuses displayed similar facial particularities consisting of macrocephaly, malar hypoplasia, small nose with anteverted nostrils and microretrognathism. Neither skeletal nor visceral anomalies were identified in the first fetus. Conversely, the second fetus presented bilateral clubfoot and severe amyotrophy of the lower limbs secondary to lumbar myelomeningocele. Associated visceral malformations consisted of left diaphragmatic hernia and bilateral renal agenesis. On CNS examination, brain weights were in accordance with the term despite hydrocephalus. No primary fissures were present with the exception of a dimple-shaped sylvian fissure. Olfactory bulbs and optic chiasm were present. External examination of the cerebellum and brainstem was normal. On sections passing through the mesencephalon, the AS was indiscernible. On coronal sections, the lateral ventricles were severely dilated. The third and fourth ventricles appeared to be collapsed in the second case. Histologically, the two brains had identical lesions. In the mesencephalon, the subcommissural organ was normal. The inferior colliculi were fused in the second case. AS atresia consisted of few rosettes lined by ependymal cells (Fig. 1a, b). Similar lesions were noted in the central canal of the medulla from the level of the area postrema to the level of decussation of the pyramids (Fig. 1c, d). At the supratentorial level, the internal capsule was normal and callosal fibres were present. Subependymal gray matter heterotopias were observed in both cases (Fig. 1e, f) and were made up with several cell types comprising an admixture of SOX2, nestin, vimentin, MAP2, GABA and GFAP immunoreactive cells. One of the most striking features was the abnormal morphology of the choroid plexuses which were voluminous due to hydrops of the connective tissue core (Fig. 2a, b) covered by intact basal lamina (Fig. 2c) and epithelial cells (Fig. 2d). No other lesions were observed in any of the different infra- and supratentorial brain structures analyzed. A targeted capture-sequencing panel including *L1CAM*, *MPDZ* and *CCDC88C* was performed in fetus 1-analysis *in solo* (Additional file 1). Two *CCDC88C* (NM_001080414.2) compound heterozygous variations were found: a indel in exon 22, c.3807_3809delinsACCT;p.(Gly1270Profs*53) and a deletion of exon 23, c.3967-?_c.4112-?;p.(Leu1323Argfs*10) (Fig. 3). Segregation analysis by Sanger sequencing demonstrated that the indel variation in exon 22 was maternally inherited, whereas the deletion of exon 23 was paternally inherited. These variations are not reported in gnomAD. According to ACMG classification, both variants are classified as pathogenic (class 5). Due to the presence of associated visceral malformations in the second case, whole exome sequencing (WES) was performed in the second fetus and his parents (trio analysis) but no additional causal variation for a second disorder was identified.

## Discussion

When excluding X-linked hydrocephalus which encompasses approximately 5–15% of congenital hydrocephalus cases, the frequency of non-syndromic forms in humans is very low, with an empiric recurrence risk rate ranging from < 1% to 4% [20], indicative of the rarity of autosomal recessive congenital hydrocephalus [19]. Using WES, variants in other genes have been recently reported as disease-causing. In the work by Shaheen et al. exploring 27 families with recurrent hydrocephalus, the most common etiologies were dystroglycanopathies (26%) and ciliopathies (15%) indicating that non-syndromic hydrocephalus is rare [16]. Using the same technical approaches, Jin et al. explored a series of 381 undiagnosed living patients with sporadic hydrocephalus and found that damaging de novo variations accounted for more than 17% of the cases. Five genes (*TRIM71, SMARCC1, PTEN, PIK3C, MTOR*) were found to be involved in syndromic forms of hydrocephalus, and three other genes (*FMN2, FOXJ1* and *PTCH1*) to be responsible for severe hydrocephalus with AS stenosis [9]. Similarly to families with recurrent hydrocephalus, non-syndromic hydrocephalus is rare in patients with sporadic hydrocephalus. To date, only 6 *CCDC88C* homozygous pathogenic variants have been identified in 14 patients with non-syndromic hydrocephalus from 6 unrelated families, including three frameshift, one splice site, and two nonsense mutations [4, 6, 13, 20]. Conversely, *CCDC88C* heterozygous gain-of-function variations have been reported in patients without hydrocephalus who suffered from adult-onset spinocerebellar ataxia SCA40 [10, 19].

Magnetic resonance imaging performed in seven out of the 12 patients with *CCDC88C* homozygous pathogenic variants revealed associated schizencephaly/porencephaly in three patients, along with either arachnoid cyst, small vermis or biparietal polymicrogyria in three other patients. Neuropathological examination performed in a single fetus interrupted at 21 WG revealed neither AS stenosis or atresia, nor ependymal rosettes or denudation [4]. Yet, neuropathological examination of the two fetuses allowed the detection of three major signs in favour of maldevelopment and malfunction of neuroepithelial/ependymal cells. First, the loss of ependyma in the AS with rosette formation leads to fusion of its dorsal and ventral walls and consequently to severe hydrocephalus. Second, early loss of ventricular zone integrity results in abnormal neurogenesis and migration. In the disrupted areas, radial glial cells disappear, depriving the neuroepithelial cells of scaffolds to migrate on. Consequently, the cells stay along the ventricular wall and form nodular periventricular heterotopias that are source of seizures in infants notwithstanding surgical CSF shunting. Third, late developmental disruption of the ventricular zone results in loss of multiciliated cells leading to alterations of the laminar CSF flow and hydrocephalus [8, 12]. Moreover, orientation of beating of cilia is defined by ependymal planar cell polarity (PCP) which involves translational and rotational movements of basal bodies. Basal body translational position movement depends on the primary cilium in connection with microtubules and apical junctions. Alterations in these junctions disrupt the natural barrier between the parenchyma and the CSF, leading to hydrocephalus, as observed in *MPDZ*-mutated patients [8]. MPDZ has been shown to bind directly DAPLE and to act as a scaffold which gathers DAPLE and other proteins involved in planar and apicobasal cell polarity pathways [11]. Therefore, the neuropathological similarities between the phenotypes linked to *MPDZ* and *CCDC88C* variants are not surprising [15]. DAPLE functions as a cellular “compass” for establishing and maintaining contact-triggered planar polarity through interaction with the third PDZ domain of MPDZ and the third PDZ domain of PARD3 [5, 18]. Interestingly, in a recent study including 138 patients with neural tube defect (NTD), rare heterozygous variants in the *PARD3* gene were identified and were shown to be significantly enriched in the aPKC-binding region resulting in defective tight junction formation via disrupted aPKC binding, suggesting that these deleterious variants contribute to human NTDs possibly by preventing apical tight junction formation and subsequent polarization process of the neuroepithelium [3]. Besides, during organ formation, proper Wnt signalling is a prerequisite for the activation of the PCP pathway, and it has also been shown that mutations in PCP genes may lead to congenital abnormalities other than NTD, notably heart, lung and kidney developmental defects. Noteworthy, in human foetuses, DAPLE expression is high in the brain and in the kidneys [6, 7]. Moreover, incomplete development of the diaphragm in the second fetus could also result also from Wnt signalling disruption, which could lead to defects in proliferation/survival of myogenic progenitor cells and defects in muscle morphogenesis by affecting extensive convergent migration of myogenic precursors during early embryogenesis. At last, our findings are in line with what already observed in MPDZ syndromic forms in which eye anomalies (chorioretinal coloboma), thoracic malformations (atrial septal defect and congenital diaphragmatic hernia) have been described and also very likely result from PCP disturbances.

## Conclusion

Multifocal atresia-forking along the AS and of the central canal of the medulla associated with periventricular neuronal heterotopias and disruption of the choroid plexus epithelium are the key hallmarks of variations in the *CCDC88C* gene, whether these brain lesions are isolated or associated with malformations suspected to result from PCP disorders. Variations in the *CCDC88C* gene together with those previously identified in the *MPDZ* gene represent a new group of emerging diseases causing congenital hydrocephalus.

## Supplementary Information

**Additional file 1:** Pathological and molecular methods.

## Data Availability

In the departments of Pathology and Genetics, Rouen University Hospital F76000, Rouen, France; in the department of Pathology at Brest University Hospital F29609, Brest, France.

## Abbreviations

AS: Aqueduct of Sylvius
CGH: Comparative genomic hybridization
CSF: Cerebrospinal fluid
CNS: Central nervous system
NGS: Next generation sequencing
NTD: Neural tube defects
PCP: Planar cell polarity
TOP: Medical termination of the pregnancy
WES: Whole exome sequencing
WG: Weeks of gestation
