# Supplementary material for: Neuropathological hallmarks of fetal hydrocephalus linked to CCDC88C pathogenic variants
Source: Acta Neuropathol Commun. 2021 Jun 6;9:104. doi: 10.1186/s40478-021-01207-5 (PMC8183048; doi:10.1186/s40478-021-01207-5)
Supplement: Supplementary file 1 — Additional file 1: Pathological and molecular methods. [file 40478_2021_1207_MOESM1_ESM.docx]

**Post-mortem examination**

*Autopsy procedures*

A complete autopsy was performed in the two foetuses (II.1 and II.2; Figure 2) with the informed written consent of the parents in accordance with the French law and following standardized protocols. Foetal biometric data were evaluated according to Guilhard-Costa et al. [5].

*Neuropathological evaluation*

Foetal brains were fixed in a 10% formalin-zinc buffer solution for one month. Brain growth and macroscopic assessment of brain maturation including gyration were evaluated according to the criteria of Guihard-Costa and Larroche and the atlas of Feess-Higgins and Larroche respectively [3, 4]. Eight-micrometer sections obtained from paraffin-embedded tissues were stained using Haematoxylin-Eosin.

Immunohistochemical studies were performed using antibodies directed against vimentin (diluted 1:100; Dakopatts, Trappes, France), glial fibrillary acidic protein (GFAP, 1:300; Dakopatts), nestin (rabbit polyclonal, diluted 1:100, Millipore, Molsheim, France), GABA (Rabbit polyclonal, diluted 1/100; Thermofisher Scientific, F67403 Illkirch Cedex, france), MAP2 (diluted 1:100; Sigma Aldrich) and SOX2 (diluted 1:.100, Abcam, Paris, France). Immunohistochemical procedures included a microwave pre-treatment protocol to aid antigen retrieval (pretreatment CC1 kit, Ventana Medical Systems Inc, Tucson AZ). Incubations were performed for 32 minutes at room temperature using the Ventana Benchmark XT system. After incubation, slides were processed by the Ultraview Universal DAB detection kit (Ventana).

**Genetic analyses**

*DNA extraction*

The parents provided written informed consent for targeted sequencing and Whole Exome Sequencing. Genomic DNA was extracted from blood samples of the parents with standard procedures and from tissue samples of the two foetuses II.1 and II.2 (Figure 2) using DNeasy Blood & Tissue kit (Qiagen, Courtaboeuf, France).

*Targeted gene panel for non-syndromic hydrocephalus*

Sequencing libraries were prepared as previously described [6]. Briefly, the capture of the 3 validated genes involved in non-syndromic hydrocephalus, *L1CAM*, *MPDZ* and *CCDC88C* was designed using Sure Design (Agilent Technologies Inc., Santa Clara, California, USA). NGS library preparations and target enrichment were performed with the SureSelect QXT kit (Agilent Technologies Inc., Santa Clara, California, USA). The multiplexed samples were sequenced on the Illumina Miseq platform (Illumina, San Diego, California, USA) using 150-bp paired-end reads.

*Whole exome sequencing*

High quality genomic DNA was sheared with a Covaris E220 DNA Sonicator (Covaris, Inc., Woburn, MA, USA), coding regions and intron-exon boundaries were captured using a SureSelectXT Human All Exon V7 kit (Agilent Technologies) according to the manufacturer’s instructions. The enriched libraries were sequenced on a NextSeq500 system (Illumina) with 2x75 bp paired-end reads

*Panel and Exome Variant analysis and prioritization*

Output paired-end sequencing data were processed for the detection of SNVs (single nucleotide variant), indels and CNV (copy number variation), through a double bioinformatics pipeline. (i) the bcl2fastq conversion software (Illumina, v2.20) was used for reads demultiplexing and generation of Fastq files. Sequenced reads were mapped to the human reference sequence (GRCh37, Hg19) using the Burrows-Wheeler Aligner (BWA v.0.7.17). Read duplicates were marked with Picard tools (v2.18.0), local realignments around indels, base-quality-score recalibration and variant calling were performed with the Genome Analysis Toolkit (GATK 4.0.6.0). Single-nucleotide variants and small indels were identified with the GATK HaplotypeCaller (v4.0.6.0), VarScan2 (v2.4.3) and Vardict (v1.5.1). Variants were then annotated with SnpEff (v.4.2) and Alamut-batch (v.1.12). (ii) In the the second pipeline, large-scale rearrangements and the related CNVs were detected using the CANOES software [1]. Variants with a minor allele frequency (MAF) of less than 1% in available were visualized on Alamut Visual software (Interactive Biosoftware) and classified in 5 classes from 1-benign to 5-pathogenic, based on MAF, predicted effect on protein, predicted effect on splicing, databases of pathogenic variations, according to ACMG recommendations.

*Sanger Dideoxy Terminator Sequencing*

The indel variant in exon 22 was confirmed by Sanger sequencing in probands and segregation analysis was performed in asymptomatic parents (primers available upon request). Sequencing products were resolved on an ABI3130xl capillary sequencing instrument (Applied Biosystems, Courtaboeuf, France).

*Digital Droplet PCR (ddPCR)*

**Universal ddPCR assay for relative was performed in the QX200 plateform (Biorad) as already described [2]. Target region into the CCDC88C gene exon 23 was amplified using the forward (5’- TGGTACTGCTCCTTGTTCTCC-3’) and the reverse (5’- AGCCAGATCCAGCTGTTGAG -3’) primer associated with the UPL50 FAM labelled LNA probe (Roche Diagnostics, USA). Reference region, in the housekeeping gene HMBS (11q23), was amplified using the forward (5’-GGGACAGTGTACCCAAGGTC-3’) and the reverse (5’-CTGAGGTAAACGGATCTGACG-3’) primer associated with a 20 base VIC labelled hydrolysis probe (5’-CCAAGAGGCTGAGGCTGAGCAGGACT-3’).**

*Bioinformatic Resources*

Primers for Sanger sequencing were designed with Primer3 software (<http://bioinfo.ut.ee/primer3-0.4.0/>). MAF was obtained on ExAC database (<http://exac.broadinstitute.org/>), EVS (<http://evs.gs.washington.edu/EVS/>), gnomAD (https://gnomad.broadinstitute.org/) and 1000 genomes (<http://www.1000genomes.org/>). Two databases of pathogenic variations, HGMD (<http://www.hgmd.cf.ac.uk/ac/index.php>) and ClinVar (<http://www.ncbi.nlm.nih.gov/clinvar/>), were consulted.

**Supplementary references**

1. Backenroth D, Homsy J, Murillo LR, Glessner J, Lin E, Brueckner M, et al (2014) CANOES: detecting rare copy number variants from whole exome sequencing data. Nucleic Acids Res. doi: 10.1093/nar/gku345.

2. Cassinari K, Quenez O, Joly-Hélas G, Beaussire L, Le Meur N, Castelain M, et al (2019) A Simple, Universal, and Cost-Efficient Digital PCR Method for the Targeted Analysis of Copy Number Variations. Clin Chem. doi: 10.1373/clinchem.2019.304246.

3. Feess-Higgins A, Larroche JC (1987) Development of the human fetal brain. Paris: (Masson eds) INSERM CNRS.

4. Guihard-Costa AM, Larroche JC (1990) Differential growth between the fetal brain and its infratentorial part. Early Hum Dev. doi: 10.1016/0378-3782(90)90126-4.

5. Guihard-Costa AM, Ménez F, Delezoide AL (2002) Organ weights in human fetuses after formalin fixation: standards by gestational age and body weight. Pediatr Dev Pathol. doi: 10.1007/s10024-002-0036-7.

6. Saugier-Veber P, Marguet F, Lecoquierre F, Adle-Biassette H, Guimiot F, Cipriani S, et al (2017) Hydrocephalus due to multiple ependymal malformations is caused by mutations in the MPDZ gene. Acta Neuropathol Commun. doi: 10.1186/s40478-017-0438-4.
